# Does ear endoscopy provide advantages in the outpatient management of open mastoidectomy cavities?

**DOI:** 10.1371/journal.pone.0191712

**Published:** 2018-01-25

**Authors:** Gustavo Subtil Magalhães Freire, Andre Luiz Lopes Sampaio, Rafaela Aquino Fernandes Lopes, Márcio Nakanishi, Carlos Augusto Costa Pires de Oliveira

**Affiliations:** Department of Otolaryngology, Hospital Universitário de Brasília (HUB), Brasília, DF, Brazil; Alexandria University Faculty of Medicine, EGYPT

## Abstract

**Objective:**

To evaluate the use of ear endoscopy in the postoperative management of open mastoidectomy cavities, and to test whether ear endoscopy improves inspection and cleaning compared with ear microscopy.

**Methods:**

Prospective study. Thirty-two ears were divided into two groups: group 1, examination and cleaning of mastoid cavities under endoscopic visualization after microscopic standard ear cleaning; group 2, examination and cleaning of mastoid cavities under microscopic visualization after endoscope-assisted ear cleaning. We assessed the ability of each method to provide exposure and facilitate cleaning, comparing the benefits of microscopy and endoscopy when used sequentially and vice-versa.

**Results:**

Endoscopy provided additional benefits for exposure in 61.1% of cases and cleaning in 66.7%. Microscopy provided no additional benefits in terms of exposure in any case, and provided added benefit for cleaning in only 21.4% of cases.

**Discussion:**

For outpatient postoperative care of open mastoidectomy cavities, ear endoscopy provides greater benefit over ear microscopy than vice-versa. In over half of all cases, endoscopy was able to expose areas not visualized under the microscope. Furthermore, in two-thirds of cases, endoscopy enabled removal of material that could not be cleared under microscopy. Ear endoscopy was superior to microscopy in terms of enabling exposure and cleaning of hard-to-reach sites, due to its wider field of vision.

**Conclusion:**

Ear endoscopy is a feasible technique for the postoperative management of open mastoidectomy cavities. Ear endoscopy provided superior advantages in terms of exposure and aural cleaning compared with microscopy.

## Introduction

Since the very first reports of endoscopic examination of the ear in 1967 [1], the use of endoscopes in otology has become progressively more common. At least two decades ago, endoscopy was introduced to otologic surgery as an adjunctive tool in the treatment of patients with cholesteatoma [2–5]. The role of ear endoscopy has since grown further, to the point where it is now considered an instrument of choice by some surgeons [2,6,7]. Its main advantage is the wide field of view, which enables visualization of areas that cannot be accessed by the operating microscope [8]. This provides a better understanding of the anatomy of the middle ear and better management of middle ear pathologies.

Despite greatly increased interest in otoendoscopic surgery in recent years, there is a lack of literature on the outpatient use of ear endoscopy for postoperative care. Whether its potential benefits are real has yet to be determined. The importance of postoperative follow-up after otologic surgery is widely known, especially after canal wall down mastoidectomy performed for cholesteatoma. On average, most patients with open mastoidectomy cavities will require 12 visits for exploration and cleaning every 5 years [9]. Despite its limitations, ear microscopy is the standard method for this postoperative care [10]. Ear endoscopy could be a useful alternative, but its role in the management of these patients is not yet well established.

**Fig 1:**
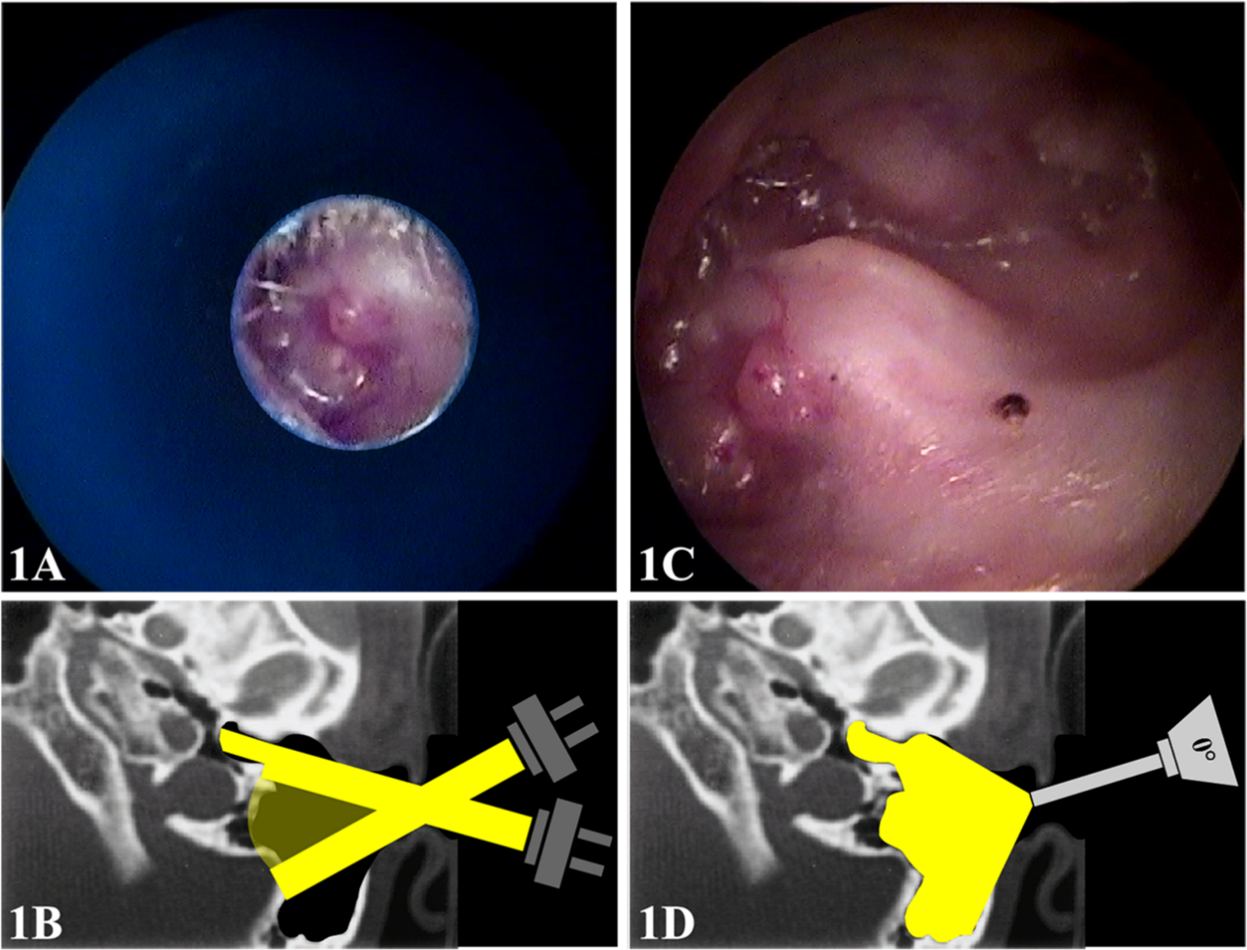
Otoscopy of a left open mastoidectomy cavity under microscopic (**1A**/**1B**) and endoscopic (**1C**/**1D**) visualization.

The wide field of view of endoscopes enables a more comprehensive examination of the mastoid cavity, even when considering narrowing of the ear canal (Fig 1). Furthermore, it can provide an improved understanding of the anatomy and physiology of the ear. Ear endoscopy can be performed with scopes used for nasal examination; no purchase of additional equipment is required. Endoscopy recording systems are widely available and play an important role in physician and patient education. Patients with a better understanding of their condition tend to be more treatment-adherent [11,12].

Despite the benefits described, additional assessment of ear endoscopy is still necessary to determine its actual role in postoperative follow-up. In this setting, endoscopy could represent an alternative tool for patient assessment or even change the way in which follow-up is conducted. Moreover, routine use in follow-up would be a good way of training otolaryngologists in the endoscopic management of the ear.

The present study sought to assess the use of ear endoscopy in the postoperative management of open mastoidectomy cavities and to test whether ear endoscopy improves inspection and cleaning capabilities compared with ear microscopy.

## Materials and methods

This prospective study was conducted at the outpatient ENT clinic of a tertiary teaching hospital. Patients who were receiving periodic follow-up at the clinic were invited to take part in the study and provided written informed consent for participation. Data collection took place from January to July 2014. This study was approved by the Universidade de Brasília School of Medicine Research Ethics Committee.

The inclusion criteria were: age 18–70 years; history of canal wall down tympanomastoidectomy performed at least 60 days before enrollment; and presence of crust/debris/discharge in the mastoid cavity and/or middle ear requiring removal. The exclusion criterion was presence of surgical complications. In patients who underwent bilateral operation, only the ear that was operated first was included in the sample.

Patients were divided into two groups. Over 3 consecutive months, all patients who presented spontaneously to the clinic for visits, were eligible, and agreed to take part in the study were allocated into group 1. The same procedure was then followed for group 2.

Ear microscopy was performed with patients in the lying position, using a light microscope (f = 250 mm). Ear endoscopy was performed with patients in the seated position using a 4-mm, 165-mm, 0° Karl Storz™ scope, endoscopy set with camera and 250W halogen light source. Forceps and suction tips were those used routinely for ear microscopy procedures.

**Fig 2:**
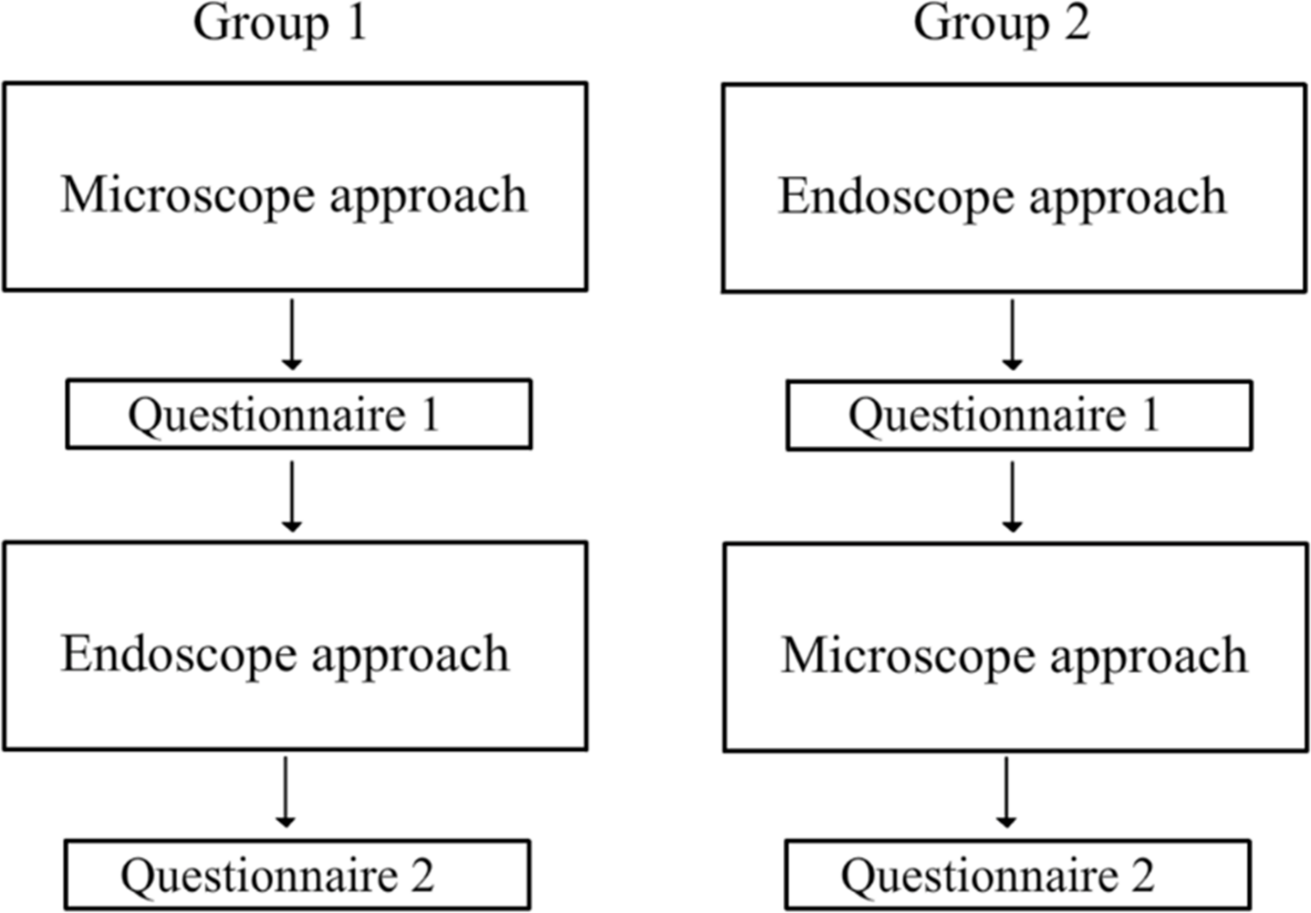
Study design.

In group 1, patients received standard care, which consisted of microscopic exploration and cleaning performed by an examiner (an otology fellow) blinded to the study objectives. Fig 2 provides an overview of the study design. This examiner completed Questionnaire 1, which was designed to collect data on the presence of material in the open cavity and the examiner’s ability to expose and clean the cavity (see S1 Appendix, which shows the questionnaire card presented to the first examiner).

Each patient was then assessed by a second examiner (a rhinology fellow), who performed endoscopic exploration. If there was any remaining material in the cavity, additional cleaning was performed under endoscopic visualization. The entire endoscopy procedure was recorded. Both fellows were recently graduated ENT specialists who worked in a subspecialty area of the study hospital as part of their respective fellowship programs. Thus, both had similar skills and experience.

The second examiner completed Questionnaire 2, which was designed primarily to assess exposure, presence of remaining material in the cavity, and cleaning ability (see S2 Appendix, which shows the questionnaire presented to the second examiner).

Regarding the amount of material requiring removal, the following semiquantitative scale was used: large amount–over 50% of the cavity filled with material; moderate amount– 25% to 50% of the cavity filled with material; small amount–less than 25% of the cavity filled with material.

The opposite order was followed by group 2 subjects, who first underwent endoscopic exploration and cleaning (performed by the rhinology fellow) followed by microscopic examination (performed by the otology fellow), using the same questionnaires.

For analysis of results (exposure and cleaning), we considered the benefits provided by the second method in relation to the first. Therefore, in group 1, we assessed whether endoscopy was able to improve exposure or cleaning after the microscope approach. For instance, when complete exposure and cleaning were achieved under microscopic visualization followed by complete exposure and cleaning under endoscopic visualization, we concluded that the second method provided no benefit in relation to the first. When exposure was considered incomplete under microscopy and complete with endoscopy, we regarded the second method as beneficial in relation to the first. When the first examiner graded cleaning as complete but the second examiner found remnants of material requiring removal, we concluded that the second method provided added benefit in terms of both exposure and cleaning (see S3 Appendix, which illustrates one such case).

The same analysis was performed in group 2, following the proper order of examiners and questionnaires. The results obtained in the two groups were then compared in an attempt to find between-group differences. We also assessed the impact of the variables of interest (size of meatoplasty, amount of material requiring removal, and difficulty of cleaning) on the results.

### Statistical analysis

All variables of interest were categorical. We tested whether each variable (size of meatoplasty, amount of material, difficulty of cleaning) impacted the primary outcomes (benefit for exposure and benefit for cleaning). We also tested for differences between groups in each variable, as well as for independence between two crossed variables, using Fisher’s exact and chi-squared tests. All tests were performed in SPSS 20.0.

## Results

The study sample comprised 32 patients. Age ranged between 18–70 years (mean, 39.1 years). Group 1 comprised 18 patients (11 male, 7 female), whereas group 2 comprised 14 ears (6 male, 8 female). Overall, there were 12 right and 6 left ears in group 1 and 8 right and 6 left ears in group 2. There were no significant between-group differences in these variables. No patient was excluded from the study.

Examiner findings regarding meatoplasty size, amount of material requiring removal, and technical difficulty are available as supplemental data (Table 1). Again, there were no significant between-group differences regarding these variables (meatoplasty, *p* = 0.1812; amount of material, *p* = 0.2071; technical difficulty, *p* = 0.1289).

**Table 1:** Size of meatoplasty, amount of secretion, and difficulty in cleaning.

		Group					
		1		2		Total	
		*n*	%	*n*	%	*n*	%
Meatoplasty	Narrow	5	27.8	7	50.0	12	37.5
	Wide	10	55.6	7	50.0	17	53.1
	Very wide	3	16.7	0	0.0	3	9.4
	Total	18	100.0	14	100.0	32	100.0
Pearson chi-square tests						*p*	0.1812
Amount of material (crust/debris/discharge)	Large	6	33.3	9	64.3	15	46.9
	Moderate	6	33.3	3	21.4	9	28.1
	Small	6	33.3	2	14.3	8	25.0
	Total	18	100.0	14	100.0	32	100.0
Pearson chi-square tests						*p*	0.2071
Difficulty cleaning	Major	1	5.6	2	14.3	3	9.4
	Moderate	3	16.7	6	42.9	9	28.1
	Minor	14	77.8	6	42.9	20	62.5
	Total	18	100.0	14	100.0	32	100.0
Pearson chi-square tests						*p*	0.1289

In group 1 (microscopy before endoscopy), endoscopic examination provided exposure benefits in 61.1% of cases (11/18). Therefore, in over half of patients, the endoscope was able to expose areas not visualized under microscopy. In group 2 (endoscopy before microscopy), subsequent microscopic examination did not provide exposure benefits in any case (0/14). Comparison of results between the groups (Table 2) showed a significant difference (*p <* 0.0002).

**Table 2:** Benefit provided by the second method after exploration and cleaning with the first method.

		Group					
		(1) Endoscopy		(2) Microscopy		Total	
		*n*	%	*n*	%	*n*	%
	No	7	38.9	14	100.0	21	65.6
Exposure benefit	Yes	11	61.1	0	0.0	11	34.4
	Total	18	100.0	14	100.0	32	100.0
Fisher’s exact test						*p*	<0.0002*
	No	6	33.3	11	78.6	17	53.1
Cleaning benefit	Yes	12	66.7	3	21.4	15	46.9
	Total	18	100.0	14	100.0	32	100.0
Fisher’s exact test						*p*	0.013*

*Significant at the 5% level.

Also in group 1, endoscopy provided benefits for cavity cleaning in 66.7% of cases (12/18); again, in half of cases, endoscopy enabled removal of material not cleared under microscopic visualization. This figure refers both to cases in which the material was not visualized under the microscope and those in which the examiner detected material but was technically unable to remove it.

In group 2, subsequent assessment under microscopy provided benefits for cleaning in 21.4% of cases (3/14). Comparison between groups showed a significant difference (*p* = 0.013). In other words, endoscopy provided more benefit over microscopy than vice-versa (Table 2).

Finally, we tested whether each of the variables of interest influenced exposure benefit and cleaning benefit results. The only variable significantly associated with primary outcomes was technical difficulty (p = 0.012 for exposure and p = 0.05 for cleaning). Greater difficulty in cleaning was associated with less exposure benefit and cleaning benefit (Table 3).

**Table 3:** Associations between benefit in exposure/cleaning and the variables meatoplasty size, amount of material present in cavity, and difficulty cleaning.

		Exposure benefit						Cleaning benefit					
		No		Yes		Total		No		Yes		Total	
		*n*	%	*n*	%	*n*	%	*n*	%	*n*	%	*n*	%
Meatoplasty	Narrow	8	38.1	4	36.4	12	12.0	5	29.4	7	46.7	12	12.0
	Wide	11	52.4	6	54.5	17	17.0	10	58.8	7	46.7	17	17.0
	Very wide	2	9.5	1	9.1	3	3.0	2	11.8	1	6.7	3	3.0
	Total	21	100.0	11	100.0	32	100.0	17	100.0	15	100.0	32	100.0
Pearson chi-square tests						*p*	0.993					*p*	0.584
Amount of material	Large	11	52.4	4	36.4	15	15.0	9	52.9	6	40.0	15	15.0
	Moderate	6	28.6	3	27.3	9	9.0	4	23.5	5	33.3	9	9.0
	Small	4	19.0	4	36.4	8	8.0	4	23.5	4	26.7	8	8.0
	Total	21	100.0	11	100.0	32	100.0	17	100.0	15	100.0	32	100.0
Pearson chi-square tests						*p*	0.531					*p*	0.745
Difficulty cleaning	Major	3	14.3	0	0.0	3	3.0	0	0.0	3	20.0	3	3.0
	Moderate	8	38.1	1	9.1	9	9.0	7	41.2	2	13.3	9	9.0
	Minor	10	47.6	10	90.9	20	20.0	10	58.8	10	66.7	20	20.0
	Total	21	100.0	11	100.0	32	100.0	17	100.0	15	100.0	32	100.0
Pearson chi-square tests						*p*	0.012*					*p*	0.05*

*Significant at the 5% level.

## Discussion

The results of this study demonstrate that, for outpatient postoperative care of open mastoidectomy cavities, ear endoscopy provides greater benefit over ear microscopy than vice-versa. In over half of all cases (61.1%), endoscopy was able to expose areas not visualized under the microscope. Furthermore, in two-thirds of cases (66.7%), endoscopy enabled removal of material that could not be cleared under microscopy. Endoscopic exploration and cleaning of open mastoidectomy cavities was thus more effective and efficient than the current standard of care (microscopy). Ear endoscopy could replace microscopy without detriment in the majority of situations. Furthermore, ear endoscopy was superior to microscopy in terms of enabling exposure and cleaning of hard-to-reach sites, due to its wider field of vision.

Endoscopy allows the examiner to transfer the source of light into the cavity, despite the small size of the ear canal. When combined with the wide field of vision, this enables broader, global visualization, including of otherwise inaccessible recesses and corners. Conversely, microscopy uses a light source projected from outside the canal. Therefore, illumination is greatly limited by the external opening of the ear canal and by the meatoplasty. Contrary to what one might speculate, the limitation of having only one hand free to manipulate the cavity under endoscopy did not cause any significant impairment. The wider view of the cavity enabled adequate manipulation of the material even with one hand only.

We did not assess whether the advantages provided by each method were associated with any clinical benefit (e.g., reduction in symptoms or flares). The purpose of this study was to assess the applicability of ear endoscopy to procedures already performed routinely under microscopic visualization. The persistence of debris in the mastoid cavity can facilitate persistence of infection. In these cases, the better removal of debris provided by ear endoscopy could contribute to better clinical control of these patients.

The fact that the first examiner was aware that the second examiner would conduct a new assessment (despite having no knowledge of the study design or purpose) could be a source of bias. The first examiner might, for instance, be less meticulous in his cleaning due to the knowledge that a second procedure would be performed. Instead, this caused a Hawthorne effect: as the first examiner knew his work would be assessed by the second, he will have tended to perform a more rigorous cleanout, thus minimizing potential bias.

The results of ear endoscopy could have been even superior if better equipment had been used. The endoscope itself was a standard nasal examination scope (diameter 4 mm, length 165 mm, 0° angulation), a model widely available at ENT clinics. Forceps and suction tips were those used routinely for ear microscopy. In cases in which not all debris or discharge could be removed under endoscopy, difficulty manipulating the forceps within the same space occupied by the scope was often the cause. This could be minimized by using narrower wide-field endoscopes, as is already the practice of some otologists, and forceps designed specifically for endoscopy. This would provide more space for manipulation without detriment to the field of view.

One limitation of this study is that, although both fellows were recently graduated ENT specialists with similar skills and experience, the second group was assessed by examiners with 3 months of additional experience. Future studies could avoid this limitation by using an examiner cross-over design and involving a greater number of fellows.

There is a lack of studies on the outpatient use of ear endoscopy for postoperative clinical care. Despite the growth of ear endoscopy in recent decades [2–5], its routine use still faces resistance from many otologists; therefore, there is no incentive to train ear surgeons in endoscopy. In view of this, some investigators have called into question how microscopy and its limitations have dictated clinical perception and management of ear conditions. The wider view provided by endoscopy has improved our understanding of diseases of the ear and how they progress in the temporal bone [2].

This is the first study to assess the outpatient application of otoendoscopy in clinical care of patients with open mastoidectomy cavities. Ear endoscopy requires training–an aspect to be tackled by ear surgeons. Its routine use for outpatient postoperative management could be a way of ensuring continuous training, thus helping with the learning curve and providing benefits for surgeons and patients alike.

## Conclusion

We conclude that ear endoscopy is an effective tool for postoperative care of open mastoidectomy cavities. In this study, ear endoscopy provided superior exposure and cleaning compared with microscopy. Ear endoscopy might thus be used for these procedures with results at least similar to those obtained with microscopy, and should be used as a supplemental technique whenever feasible.

## Supporting information

S1 Appendix Questionnaire presented to first examiner. (TIF)

S2 Appendix Questionnaire presented to second examiner. (TIF)

S3 Appendix Representative endoscopic procedure. (TIF)
