# Supplementary figures and images for: Does ear endoscopy provide advantages in the outpatient management of open mastoidectomy cavities?
Source: PLoS One. 2018 Jan 25;13(1):e0191712. doi: 10.1371/journal.pone.0191712 (PMC5784996; doi:10.1371/journal.pone.0191712)

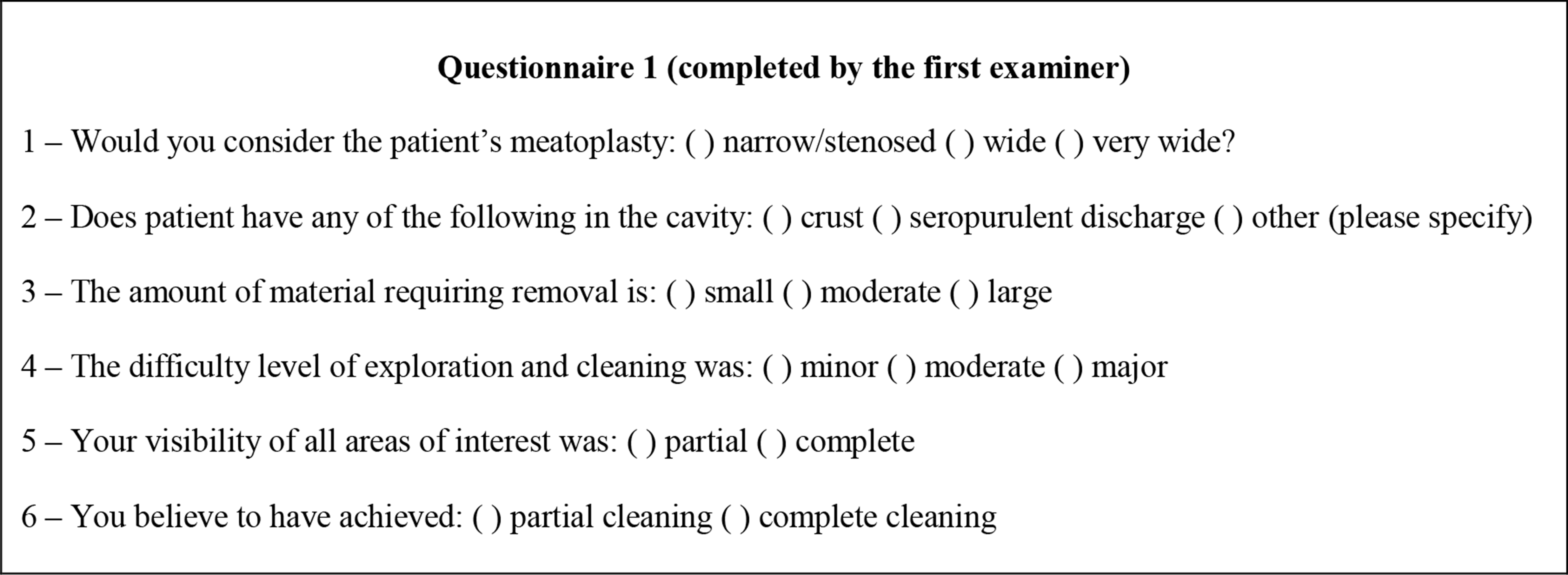

Supplement: S1 Appendix — (TIF) [file pone.0191712.s001.tif]

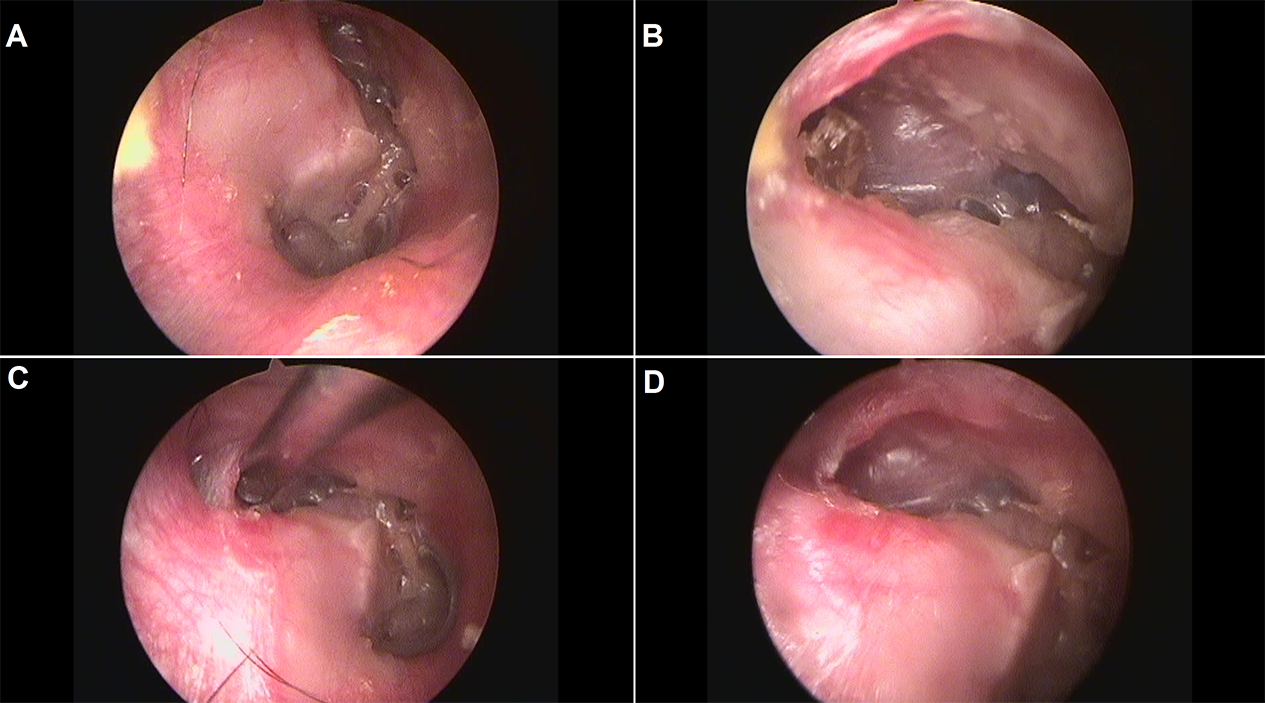

Supplement: S3 Appendix — (TIF) [file pone.0191712.s003.tif]
